# Using a Computerised Staircase and Incremental Optotype Sizes to Improve Visual Acuity Assessment Accuracy

**DOI:** 10.22599/bioj.271

**Published:** 2022-07-20

**Authors:** Anna O’Connor, Chloe King, Ashli Milling, Laurence Tidbury

**Affiliations:** 1 University of Liverpool, GB

**Keywords:** Visual acuity, vision tests, test-retest variability

## Abstract

**Background:**

Given the impact of visual acuity results on diagnosis and management, it is essential that the test is accurate, determined by factors such as test-retest variability. Standardisation improves accuracy, which can be performed via a computerised staircase methodology. Standard clinical tests with scoring of 0.02 per optotype implies an incremental score per optotype despite optotype size remaining constant on each line. The aim of this study is to establish if near continuous incremental optotype display and scoring improves test-retest variability compared to current testing methods.

**Methods:**

A computerised three up, one down adaptive staircase was used to display Kay Picture optotypes on an LCD monitor. Three methods of visual acuity assessment were undertaken: ETDRS, Kay Pictures and computerised Kay Pictures. Tests were performed twice under standard clinical conditions.

**Results:**

One hundred nineteen adults were tested. Test-retest variability for computerised Kay pictures was 0.01 logMAR (±0.04, p = 0.001). Good levels of agreement were observed for computerised Kay pictures in terms of test-retest variability, where the test had the smallest mean bias (0.01 logMAR compared to 0.03 and 0.08 logMAR for Kay Pictures and ETDRS respectively) and narrowest limits of agreement. Participants performed better in computerised Kay pictures than Kay Pictures by 0.03 logMAR, and better in ETDRS than computerised Kay pictures by 0.1 logMAR.

**Conclusion:**

Computerised Kay pictures exhibited a low test-retest variability, demonstrating it is reliable and repeatable. This repeatability measure is lower than the test-retest variability of the ETDRS and Kay Pictures tests.

Visual acuity is the ability to resolve spatial detail, assessed in all patients requiring eye care. It is crucial that the method of measuring visual acuity is accurate as it may be indicative of ocular health, and provides clinicians the ability to monitor visual abnormalities and thus, often dictates treatment plans. In the paediatric population conditions such as refractive error and amblyopia are identified through assessment of visual acuity (Garretty 2017; Powell & Hatt 2009). Their detection is important given that both uncorrected refractive error and amblyopia have been shown to negatively impact children’s learning, leading to poorer socio-economic prospects and a poorer quality of life (Birch & Kelly 2017; Hatt et al. 2020; Lamoureux et al. 2009).

Visual acuity testing methodology has gone through many evolutionary changes, often galvanised by technological advancement, and included notable developments such as the introduction of the Snellen Chart in 1862 and the logMAR principles of Bailey and Lovie (1976). The current gold standard for adult visual acuity assessment in the UK, as recommended by the International Council of Ophthalmology, is the Early Treatment for Diabetic Retinopathy Study (ETDRS) chart which is based on the logMAR design principles (International Council of Ophthalmology 1988). In contrast, in the paediatric setting there are a variety of tests utilised, dependent on the age of the child and other factors, but each test is guided by the logMAR design principles (Anstice & Thompson 2014). Visual acuity tests must have a proven ability to be accurate and reliable, demonstrated by low test-retest variability and being comparable with clinical standards. The test-retest variability can be used to determine confidence intervals to ensure that a change of visual acuity is reflective of actual change rather than as a result of chance variation. Assessment methods should also have a near absent learning effect, in order that repeat test results are not influenced by memory of previous tests, which may mask degradation of visual acuity.

Current technologies have allowed the creation of bespoke software to measure visual acuity via a computer-generated staircase measurement, with single optotype presentation. The main advantages of this methodology are further standardisation of protocols and automation of the system, minimising the impact of human bias, with results demonstrating reliable acuity measurements (Holmes et al. 2001; Moke et al. 2001; Shah et al. 2011). This protocol is particularly useful in paediatrics, as single optotypes are easier for children to identify than the lines presented in linear tests (Simmers et al. 1997).

LogMAR design principles ensure standardisation across tests, with results scored per line or per optotype. When scoring per optotype it means a different value is attributed based on the number of optotypes seen on a line, despite the optotype size being constant. For example, scores of 0.58 and 0.50 logMAR both represent recognition of the same optotype size, but different scores suggest different sizes seen (see Figure 1). Therefore, introducing an incremental score where the optotype size changes in smaller step sizes, may have the potential to improve test accuracy in terms of test-retest variability. This is supported by evidence using simulations of visual acuity testing which showed a reduction in test-retest variability by reducing the step size between lines, and increasing the number of optotypes per line (Raasch et al. 1998).

**Figure 1:**
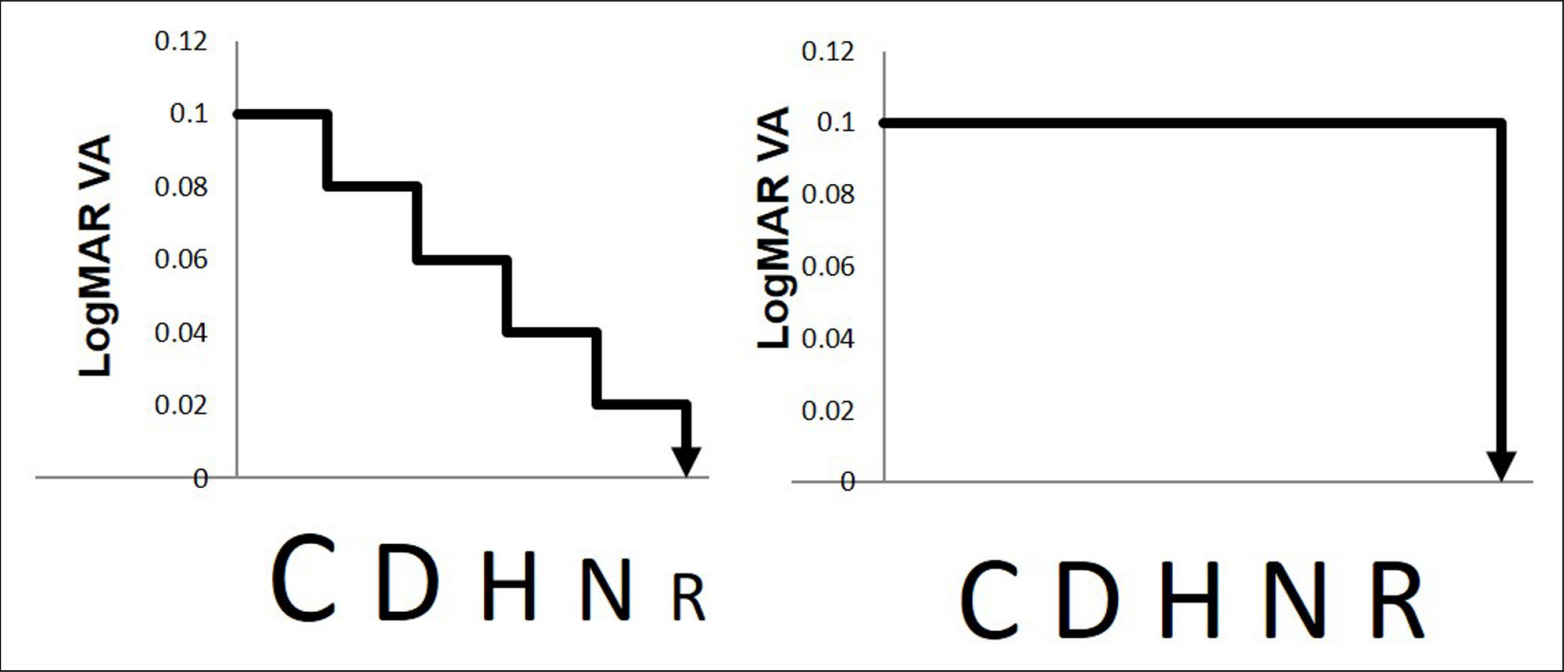
Schematic showing the incremental changes in optotype size proposed in comparison to the constant size of current VA tests.

The purpose of the current study was to combine the methods of a staircase procedure with incremental optotype sizes, evaluate the test-retest variability of this procedure using Kay Picture optotypes and compare the visual acuity measurements to current methods of testing. To evaluate the test without the potential impact of variations in cognitive ability, adults were tested.

## Methods

The study was approved by the Committee on Research Ethics at the University of xxxxxxx and informed consent was obtained prior to testing. The data sets were collected as part of an orthoptic undergraduate project, meaning that there were multiple testers, but all were experienced in the clinical assessment of visual acuity. Each participant was examined by one tester for all three methods of visual acuity assessment. The software for the staircase measurement to measure visual acuity and protocols for all visual acuity testing was standardised. Three visual acuity tests were employed to investigate the research aims: the ETDRS logMAR chart, the printed book Kay Picture test (single crowded optotype version), and the computerised Kay Picture test. All visual acuity tests were measured on the logMAR scale.

### Participants

Participants were recruited into the study from the student population at the University of XXXXXXX. The criteria for inclusion was that the participants were over the age of eighteen years. There was no upper age limit for the study. Exclusion criteria was any ocular abnormality as determined by a brief ocular history of the participant.

### Display

All charts were performed twice in a pseudorandomised order in one single session. The tests were performed under standard clinical lighting and scored per optotype on the logMAR scale. As the tests were conducted in the same room, the luminance was the same for all testing methods. Participants were assessed binocularly and wore their habitual correction during testing. Testing under binocular conditions was chosen to minimise the potential fatigue effects of doubling the number of tests.

### Protocol

#### ETDRS

The ETDRS chart was viewed on Thomson Test Chart software on an LG monitor (same as the Computer Kay Pictures) at 4 m. Participants were asked to read across, and down, the chart until the optotypes could no longer be discerned. There were five optotypes per line and the test was scored per optotype (0.02 per optotype). The termination criteria was determined as three incorrectly identified optotypes in a row. The optotypes were randomised before performing the second test.

#### Book Kay pictures

Kay Pictures were displayed in a standard book format with single crowded optotypes (same format as the computer test) and viewed at a distance of 6m. Increasing the standard test distance (3m) ensured that a true threshold would be measured with no ceiling effect, the score was adjusted to reflect this increase, with a value of 0.3 subtracted from the score indicated in the book. Participants were asked to identify the optotype displayed in decreasing sizes, with one optotype per logMAR line until it could no longer be seen and correctly identified. Termination criteria was deemed as three incorrectly identified optotypes on a line. The test was scored per optotype (0.02 per optotype).

#### Computerised Kay pictures

A one up, three down adaptive staircase procedure was coded using psychopy (Peirce 2007) to control the display of Kay pictures optotypes, with three interleaved staircases. The staircases started at an optotype height of 10, 16 and 32 pixels. Each optotype size was presented twice, if correct the following reduction was half the size of the optotype presented. The test was displayed on an HP monitor (Compaq Elite 8300), with a screen width of 50.5 cm, at 3 m. The screen had a resolution of 1920 × 1080 pixels with each pixel subtending 0.005° or 18.1 arc seconds. The screen size, resolution and distance required for the crowding box, provided the capacity to measure visual acuity between 0.505 and –0.46 logMAR. The logMAR value being based on whole pixel sizes but aliasing was utilised to create the recognisable shapes. Optotype size displayed on screen was not limited to the sizes included on a standard logMAR chart. Instead, to avoid any uncontrollable software implementation of antialiasing, (which results in varying contrasts at the edge of optotypes in order to create a smooth appearance) the displayed sizes of the optotypes were limited to whole pixels only, thus ensuring the 100% contrast required for a visual acuity test. The sizes of optotype displayed were therefore ‘continuous’, only restricted by pixel size and available screen area. In a standard logMAR chart 0.02 represents one out of five rather than a number, in this computer programme 0.02 truly reflects the angular size of the optotype (Tidbury et al. 2016).

One of the six images (from the updated Kay pictures test (Milling et al. 2016)), presented in an appropriately spaced crowding box, was shown to the participant in a randomised order. Responses were recorded by the participants using a custom push button panel. A six alternative force choice response box was given to participants, with named buttons corresponding to the pictures. The staircase procedure was terminated by the programme after eight reversals with participants required to guess when they were unable to see the optotype. The chance of guessing the optotype correctly was 0.083. Visual acuity thresholds were determined by fitting a Weibull Cumulative Distribution Function on MATLab, using the last eight reversals, weighted by the number of trials for each participant at the level of 70% correct.

#### Statistical Analysis

Data were stored and organised on Microsoft Excel for Mac, version 15.3.1, and analysed on SPSS, version 25. Descriptive analysis of the data was employed to give an overview of the study demographics and the distribution of the data, using standard deviation as the measure of variability. The methods of Bland and Altman with a 95% limit of agreement were used to visualise the data and performance was measured in terms of bias (mean difference between tests) and test-retest variability (Bland & Altman 1999). The higher and lower limits of agreement were plus and minus 1.96 standard deviations from the bias respectively. For the test-retest analysis, the data for the second test were subtracted from the first test data to calculate the difference and for the method comparison analysis, so a positive bias indicates an improvement in acuity in test two. The alternative visual acuity test data were taken from the computerised Kay pictures data. Furthermore, significance of the scores obtained were determined by paired t-tests for the test-retest and test comparison analyses.

## Results

### Study Demographics

A total of 153 participants were recruited into the study and 119 participants included in the final analysis. Participants (n = 34) were excluded due to incomplete data sets from two student groups. Eighty-seven participants (73%) were female and 32 (27%) were male. The mean (SD) age of the population was 21.5 (±5.3) years, the ages ranged from 18 to 59 years.

### Test-Retest Data

Paired t-tests showed there was no significant difference in the test and retest data for ETDRS (p = 0.08) but the difference was significant for book Kay pictures and computerised Kay pictures (p = 0.001 for both). The mean (± one standard deviation) visual acuity differences (test one – test two) are shown in Table 1.

**Table 1:** Summary of the data from the Bland-Altman plots showing comparison of test-retest data and methods of testing. For the mean difference a positive value indicates that the first test was numerically higher (worse VA) than the second.


	TEST	MEAN DIFFERENCE	STANDARD DEVIATION	UPPER LIMITS OF AGREEMENT	LOWER LIMITS OF AGREEMENT	P VALUES

**Test retest variability**	ETDRS	0.081	0.05	0.179 (0.10)	–0.017 (–0.08)	0.08

	Book Kay Pictures	0.027	0.084	0.192 (0.16)	–0.138 (–0.10)	0.001

	Computer Kay Pictures	0.012	0.039	0.089 (0.08)	–0.065 (–0.06)	0.001

**Methods Compared**	Computer Kay Pictures1 vs. ETDRS1	–0.097	0.098	0.095 (–0.02)	–0.289 (–0.21)	0.000

	Computer Kay Pictures2 vs. ETDRS2	–0.101	0.086	0.069 (–0.02)	–0.271 (–0.22)	0.000

	Computer Kay Pictures1 vs. Book Kay Pictures1	0.033	0.138	0.304 (0.22)	–0.237 (–0.13)	0.000

	Computer Kay Pictures2 vs. Book Kay Pictures2	0.034	0.129	0.286 (0.24)	–0.219 (–0.13)	0.000

	Book Kay Pictures1-ETDRS1	–0.147	0.109	0.067 (0.04)	–0.361 (–0.33)	0.000

	Book Kay Pictures2-ETDRS2	–0.166	0.117	0.063 (0.33)	–0.395 (–0.36)	0.000


**Figure 2:**
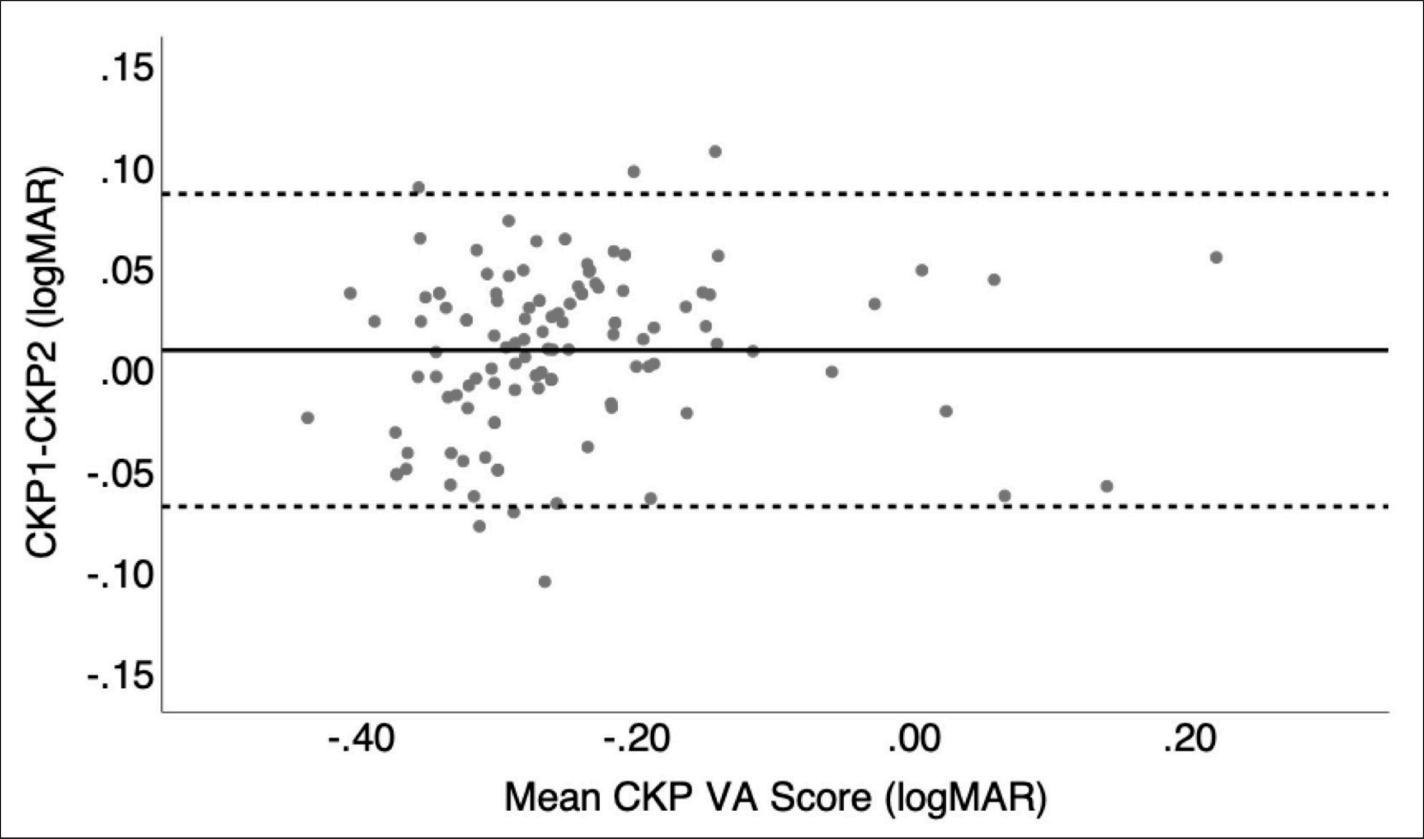
Bland-Altman plot displaying the test-retest variability for the adaptive staircase procedure where the solid line represents the mean bias and the dashed line is the upper and lower limits of agreement.

**Figure 3:**
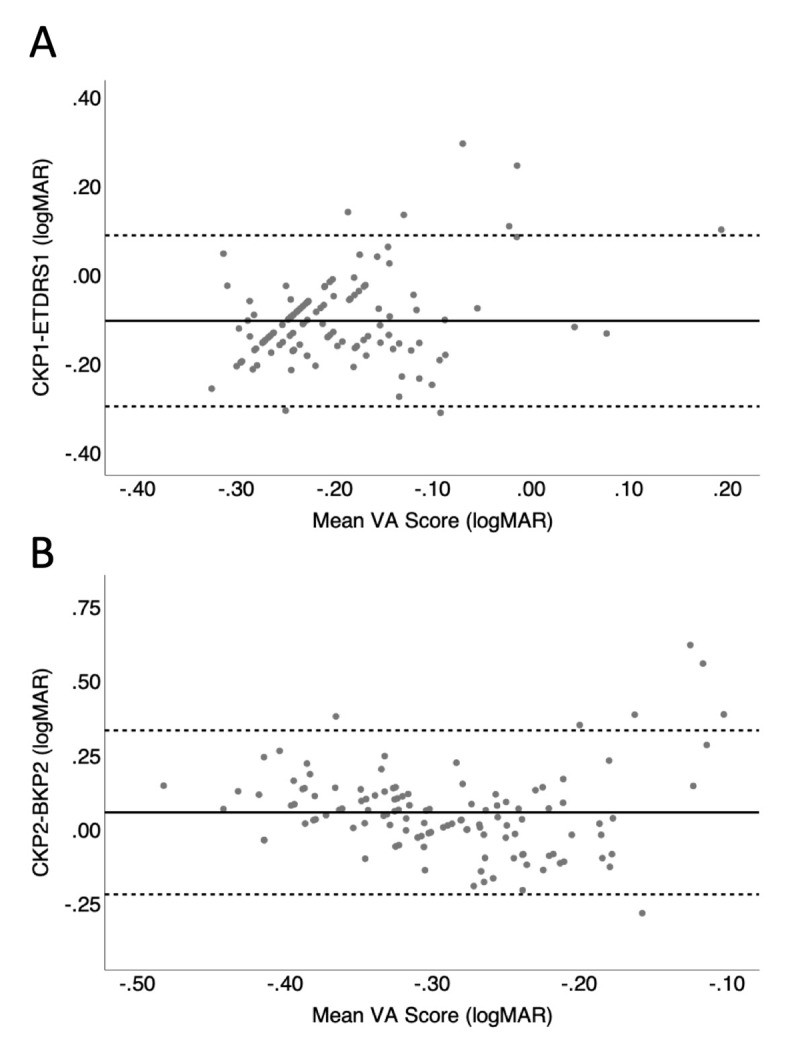
Bland-Altman plot comparing the novel Computerised Kay Pictures (computerised Kay pictures) staircase procedure with current tests. The solid line represents the mean bias and the dashed line is the upper and lower limits of agreement. A) Bland-Altman plot for computerised Kay pictures and the adult gold standard, ETDRS for the first test. B) Bland-Altman plot for computerised Kay pictures and Book Kay Pictures for the second test.

Bland Altman plots were used to assess the level of agreement in the data where the data are summarised in Table 1 and displayed in Figures 2 and 3. ETDRS had a mean bias value of 0.08 (±0.05) logMAR. All data outside the limits of agreement (n = 19) were below the lower limit.

Book Kay pictures had a difference of 0.03 (±0.08) logMAR between test one and two. There were a total of six data points outside of limits of agreement, three above the upper limits of agreement and three below the lower limits of agreement.

Computerised Kay pictures had the lowest bias level of 0.01 (±0.04) logMAR. Six data points were situated outside of the limits of agreement of which three were above the upper limit and three were below the lower limit. The data showed a positive trend between the mean score and score difference.

### Method Comparison Analysis

Bland Altman plots for the original test data for computerised Kay pictures compared to ETDRS show a negative mean bias, where participants had a numerically higher visual acuity (clinically worse score) in the ETDRS than computerised Kay pictures. The mean difference between tests and limits of agreement are summarised in Table 1. The level of difference was similar for both test and re-test data and the data were similarly distributed.

Bland Altman plots of computerised Kay pictures vs. book Kay pictures showed a negative trend where five data points were located above the upper limits of agreement and three below the lower limits of agreement. Similarly, the retest data shows a negative trend, shown in Figure 3.

## Discussion

The visual acuity results from the computerised Kay pictures data shows promise, having the smallest level of bias and smallest variability (with the narrowest limits of agreement) on the test-retest data. The optimal outcome for test-retest variability would be as close to zero as possible with narrow limits of agreement. The mean bias for computerised Kay pictures was the closest to zero of the three tests and the narrowest limits of agreement. The positive value of bias demonstrates that the visual acuity scores were marginally numerically higher (meaning a worse visual acuity score) on the original test in comparison to the retest, suggesting a slight practice effect, but the mean bias is only 0.01. As the variation of the data was smallest in the adaptive staircase procedure it was therefore superior to both the currently used adult and paediatric test in respect to consistency in the results achieved.

The current study is consistent with previous research such as the Electronic Visual Acuity Tester HOTV protocol which found similarly superior results of an adaptive staircase measurement compared to standard visual acuity testing (Moke et al. 2001). While research using the Electronic Visual Acuity Tester found a beneficial impact of using a staircase measurement, it should be noted that the research showed greater variability than the current study, although this could be due to the inclusion of participants with visual abnormalities (Drover et al. 2008). Furthermore, the Electronic Visual Acuity Tester had four optotypes (HOTV) as opposed to the six used in the current study, plus fewer reversals, which could also account for the differences in variability.

Good levels of agreement and consistent results were found in the comparison between methods. Visual acuity was numerically higher in the adaptive staircase procedure than book Kay pictures by 0.03 in the test and retest data. Whereas the comparison between computerised Kay pictures and ETDRS demonstrated, visual acuity was numerically lower in computerised Kay pictures than ETDRS (meaning the computerised Kay pictures indicated better visual acuity) by one line in both the test and retest data. Although there is some difference in measurements, it is reported that, for a standard clinical test where the optotype sizes are equal within the line, a difference of 0.2 logMAR is required to reach clinical significance when detecting a change in visual acuity (Shah et al. 2011). Given the narrower limits of agreement with computerised Kay pictures this could potentially be reduced, increasing the test sensitivity to a change in visual acuity. Furthermore, it is known that picture-based tests typically give a visual acuity value that is approximately one line difference (indicating better visual acuity) than letter-based tests, the consistency in differences across the tests allows the clinician to interpret and adjust the result accordingly (Milling et al. 2016; Moganeswari et al. 2015). Previous research has indicated that variability in visual acuity increases with poorer vision by up to two lines in stable amblyopic patients (Shah et al. 2012). This variation in acuity is in part attributable to the test-retest variability but combined with a true variation in visual acuity. As the new method has a smaller test-retest variability, it could be used to detect smaller amounts of change in acuity in patients.

These initial results are encouraging, but there were some limitations in the methodology. The protocol for the staircase measurement was terminated after eight reversals, with the amount of trials ranging from 63 to 149. This took a considerable amount of time according to anecdotal reports from participants, although the data do not demonstrate any fatigue effect, and would therefore not be suitable in terms of practicality for paediatric vision testing in its current form. However, a reduced staircase could be optimised to operate in a shorter period of time, as clinical tests often have to compromise between accuracy and logistical restraints while maintaining a sufficient degree of accuracy. Given the duration of the testing and familiarity with the optotypes, learning or fatigue effects may have occurred. However, the similarity in the distribution of the data and trends observed in the test and retest comparisons suggests that the results are a true reflection of the performance of the participant and underlying population, thus suggesting a minimal learning effect and the effectiveness of the randomisation of the tests. The use of multiple testers may have introduced another variable impacting on the outcomes, however, this is reflective of a true clinical situation. Also, it would be anticipated that this would increase the variability found, but as the ETDRS and Kay pictures test-retest variability values were similar to previous reports (Beck et al. 2003; Milling et al. 2016; Shah et al. 2011), it does not appear to have had an adverse impact.

This study was conducted in adult participants with good levels of visual acuity to evaluate the test procedure. The rationale for assessing adults rather than children was to increase the reliability of the data collected and ensure a rigorous testing of the methodology due to the known limitations of working with children, including shorter attention spans, ranging cognitive abilities and the variability in the maturity of the visual system. This methodological approach enabled us to evaluate the accuracy of the test, but expanding the study population to include children is also required for determining testability using staircase methodology within the paediatric population. In addition, a wider range of acuities and visual disorders would need to be evaluated as reduced acuity has been linked to the variability between tests in children with amblyopia (Birch et al. 2009).

## Conclusion

This study aimed to further standardise and improve a picture visual acuity assessment, by introducing smaller increments to the optotype size (that truly reflect the scores given) combined with a computerised staircase. The aforementioned advantages as well as the high test-retest reliability and superiority observed to current tests provide evidence that the protocol may be suitable for clinical use for the detection, management and research of ocular disease. However, modifications would be required in relation to reducing the time to complete the test, this could be achieved by the utilisation of a more efficient adaptive procedure.
