# Corrigendum: Using a Computerised Staircase and Incremental Optotype Sizes to Improve Visual Acuity Assessment Accuracy

**DOI:** 10.22599/bioj.287

**Published:** 2022-11-16

**Authors:** Anna O’Connor, Chloe King, Ashli Milling, Laurence Tidbury

**Affiliations:** 1 University of Liverpool, GB

**Keywords:** Visual acuity, vision tests, tes-tretest variability

## Abstract

This article details a correction to: O’Connor, A., King, C., Milling, A. and Tidbury, L., 2022. Using a Computerised Staircase and Incremental Optotype Sizes to Improve Visual Acuity Assessment Accuracy. *British and Irish Orthoptic Journal*, 18(1), pp. 93–100. DOI: http://doi.org/10.22599/bioj.271.

## Correction

The original article (O’Connor et al, 2022) was erroneously published with placeholder text that had been included during the peer review process to ensure anonymity. The name of the university where the research was carried out (the University of Liverpool) was omitted from various sections.

The affected sentences are corrected below.

## Methods

The first sentence should read:

The study was approved by the Committee on Research Ethics at the University of Liverpool and informed consent was obtained prior to testing.

## Participants

The first sentence should read:

Participants were recruited into the study from the student population at the University of Liverpool.
